# Chronic Vaginal Candidiasis Is Achievable in Outbred CD-1 Mice

**DOI:** 10.1128/mBio.01372-17

**Published:** 2017-10-24

**Authors:** Elena Gabrielli, Elena Roselletti, Eva Pericolini, Samuele Sabbatini, Anna Vecchiarelli, Antonio Cassone

**Affiliations:** a Department of Experimental Medicine, Microbiology Section, University of Perugia, Perugia, Italy; b Department of Diagnostic, Clinic and Public Health Medicine, University of Modena and Reggio Emilia, Modena, Italy; c Polo d’Innovazione della Genomica, Genetica e Biologia, University of Perugia, Perugia, Italy; Albert Einstein College of Medicine

**Keywords:** *Candida albicans*, chronic infection, mice, vaginitis

## LETTER

In their insightful paper, Yano and collaborators (1) have recently reported that outbred CD-1 mice are resistant to chronic vaginal infection (vulvovaginal candidiasis [VVC]), presumably due to an inherent nonresponsiveness to estrogen treatment (2). In view of the importance of establishing a mouse model of resistance to VVC (rVVC) in animals with the genetic heterogeneity and variable individual susceptibility to infection that also characterizes human vaginal infection (3), we examined whether rVVC could also be induced in CD-1 mice in a vis-a-vis comparison with typically VVC-susceptible C57BL/6J mice.

**FIG 1:**
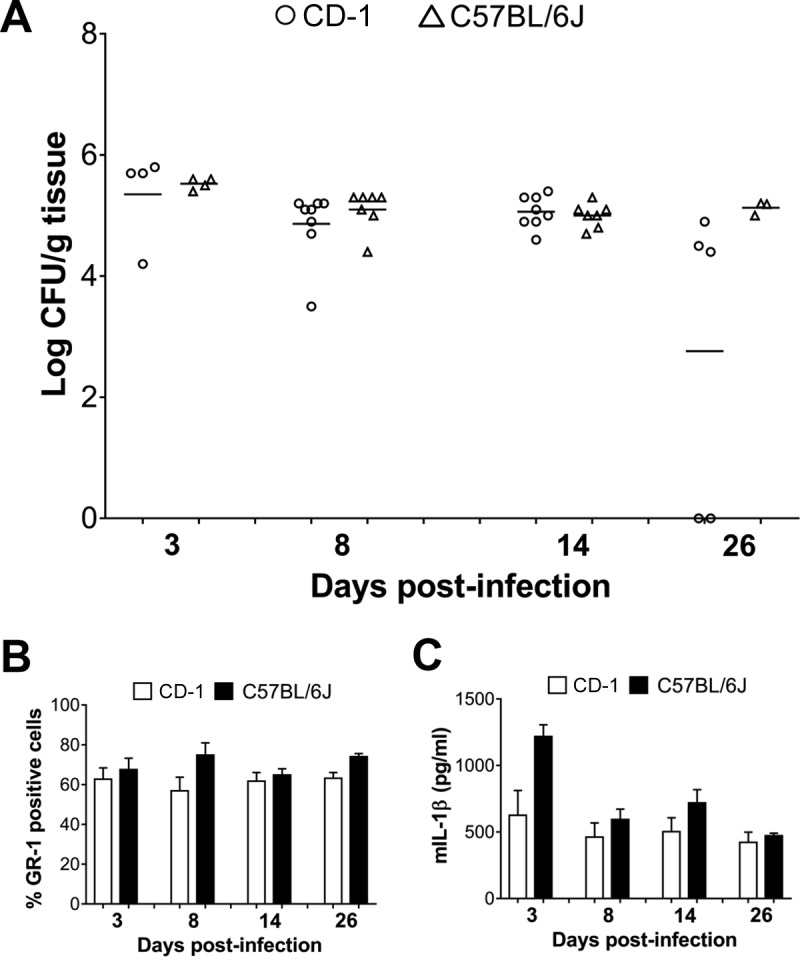
Vaginal *C. albicans* CFU counts (A) and percentages of GR-1 positive cells (B) and IL-1β concentration (C) in the vaginal fluid from CD-1 and C57BL/6J mice challenged with 2 × 10^6^ fungal cells/mouse on day 0 and monitored longitudinally for infection and inflammation at days 3, 8, and 14 in one experiment and on days 8, 14, and 26 in another experiment. In panel A, each symbol represents the value for an individual mouse A horizontal bar shows the mean CFU for a group of mice in panel A, while the values for GR-1 positive percentages (B) and IL-1β concentrations (C) are reported as means plus standard errors of the means (SEM) (error bars). For details about the fungal strain used, estrogen treatment, and methods of evaluation of infection, neutrophils, and cytokine determination, see references 4 and 5. Data were assessed for statistical significance by a two-tailed Student’s *t* test. mIL-1β, mouse IL-1β.

We initially determined infectious burden and vaginal inflammatory markers (neutrophils and interleukin-1β [IL-1β]) on different days post-infection in mice infected with different doses of *Candida albicans* (for experimental details, see reference 4 and the legend to Fig. 1). Here we confirm the data reported by Yano et al. (1) that at a relatively low challenge dose (around or below 10^5^ fungal cells), the intravaginal fungal CFU were comparable in the two strains of mice until day 3 but decreased dramatically in CD-1 mice on the following days. However, when infected by 2 × 10^7^ cells, CD-1 and C57BL/6J mice had similar degrees of infection and inflammation throughout the observation period (up to 14 days; data not shown). This suggested that the early loss of infection in the outbred mice could be due to an insufficient challenge dose. Thus, CD-1 and C57BL/6J mice were challenged with 2 × 10^6^ fungal cells, keeping all other conditions unchanged (as in previous experiments [4]), and prolonging the observation period until day 26 after challenge.

Figure 1 shows the cumulative data from two experiments. Vaginal fungus loads (Fig. 1A) were consistently and persistently high in all mice, with no significant difference between CD-1 and C57BL/6J mouse strains except on day 26, when no CFU were recovered from two of the five CD-1 mice. However, the other three mice had CFU counts quite comparable to those of the C57BL/6J mice (4.6 ± 0.12 versus 5.1 ± 0.05 [mean log CFU/g of tissue ± SEM]). Notably, both neutrophil numbers (Fig. 1B) and IL-1β concentrations in the vaginal fluid (Fig. 1C) were similar overall in the two strains of mice on each day examined, including day 26, with no statistically significant differences between them.

The experiments described above show that CD-1 mice are not inherently refractory to persistent vaginal infection and inflammation by *C. albicans*. Their reported resistance to VVC (1) can be overcome by increasing the size of the infectious inoculum, which could make the estrogen requirement less stringent (2). Mechanistically, the data obtained so far in our CD-1 model infected with high fungal inoculum are consistent with the well-established features of experimental vaginal candidiasis in typically susceptible mice: high levels of inflammatory markers, inability of the massively recruited neutrophils to clear the infection, possibly due to *Candida* or host-derived inhibitors, and role of one or more fungal aspartyl proteinases as inflammation inducers (1, 4–7).
